# Malunion of Pediatric Forearm Shaft Fractures: Management Principles and Techniques

**DOI:** 10.1007/s12178-022-09783-2

**Published:** 2022-07-25

**Authors:** T. Peter Li, Adi Wollstein, Samir Sabharwal, Suresh K. Nayar, Sanjeev Sabharwal

**Affiliations:** 1 Department of Orthopaedic Surgery, Johns Hopkins University School of Medicine, Baltimore, MD USA; 2 Department of Orthopaedic Surgery, University of California San Francisco, Benioff Children’s Hospital, Oakland, CA USA

**Keywords:** Pediatric forearm fracture malunion, Corrective osteotomy, 3D computer-assisted planning, Patient-specific instrumentation

## Abstract

**Purpose of Review:**

Clinically significant malunion of forearm diaphyseal fractures is an uncommon but potentially disabling condition amongst children and adolescents. We present the preoperative evaluation, including imaging, and discuss surgical indications and contemporary approaches to manage such patients, including an illustrative case.

**Recent Findings:**

While advances in three-dimensional (3D) simulation, modeling, and patient-specific instrumentation have expanded the surgical armamentarium, their impact on long-term outcomes compared to traditional methods remains unknown.

**Summary:**

Successful outcome following surgical correction of malunion following a both-bone forearm fracture can be achieved with careful patient selection, appropriate indications, and a well-planned surgical execution.

## Introduction

Radiographic malunion is common following closed treatment of pediatric forearm diaphyseal fractures, ranging from 15 to 39% in children who sustained the injuries up to 15 years of age [1–3]. Most children continue to remodel their deformities [4–6], reducing the incidence of radiographic malunion to 2.4–13.2% [1–3] up to 13.5 years from the time of injury [3]. Despite the prevalence of residual deformity, majority of pediatric patients with radiographic malunion do not have clinical complaints, with only 0.5% going on to symptomatic malunion [1, 2]. Moreover, patients’ perceived disability does not directly correlate with radiographic malunion [7–9]. Therefore, pediatric patients with symptomatic forearm fracture malunion who are candidates for surgical correction are rare and oftentimes difficult to diagnose. Symptomatic malunion can be painful, visually prominent and functionally disabling [4, 10–14].

There is currently no cohesive classification scheme to guide treatment or establish prognosis of pediatric forearm fracture malunion. Nevertheless, various metrics [15–18] do exist to define “unacceptable” reduction and healing of diaphyseal forearm fractures based on physical exam and imaging studies and influence treatment decision-making for these injuries.

The accepted definition of radiographic malunion remains debatable, as there is a wide variation of thresholds defined for “tolerated” deformity. In principle, surgical correction is reserved for malunions associated with functional impairment of forearm rotation, cause pain at the distal radioulnar joint (DRUJ) or create visible deformity that is unacceptable to the patient or family [14, 19, 20]. Corrective techniques have evolved in an attempt to create precise, function-restoring and near-anatomic deformity correction [10, 14, 19, 21•]

The optimal timing of surgical intervention is generally advocated within 1 year of injury [13, 20]. Uncorrected malunion can result in degenerative joint disease in the proximal and distal radio-ulnar joints [22]. Surgical options are becoming more advanced, particularly in an era of rapid technologic advancement and surgical innovation. Management principles along with various preoperative imaging and techniques employed for surgical correction of forearm malunion in the pediatric patient will be described in this review along with an illustrative case.

## Pathologic Anatomy and Indications for Surgery

**Fig. 1:**
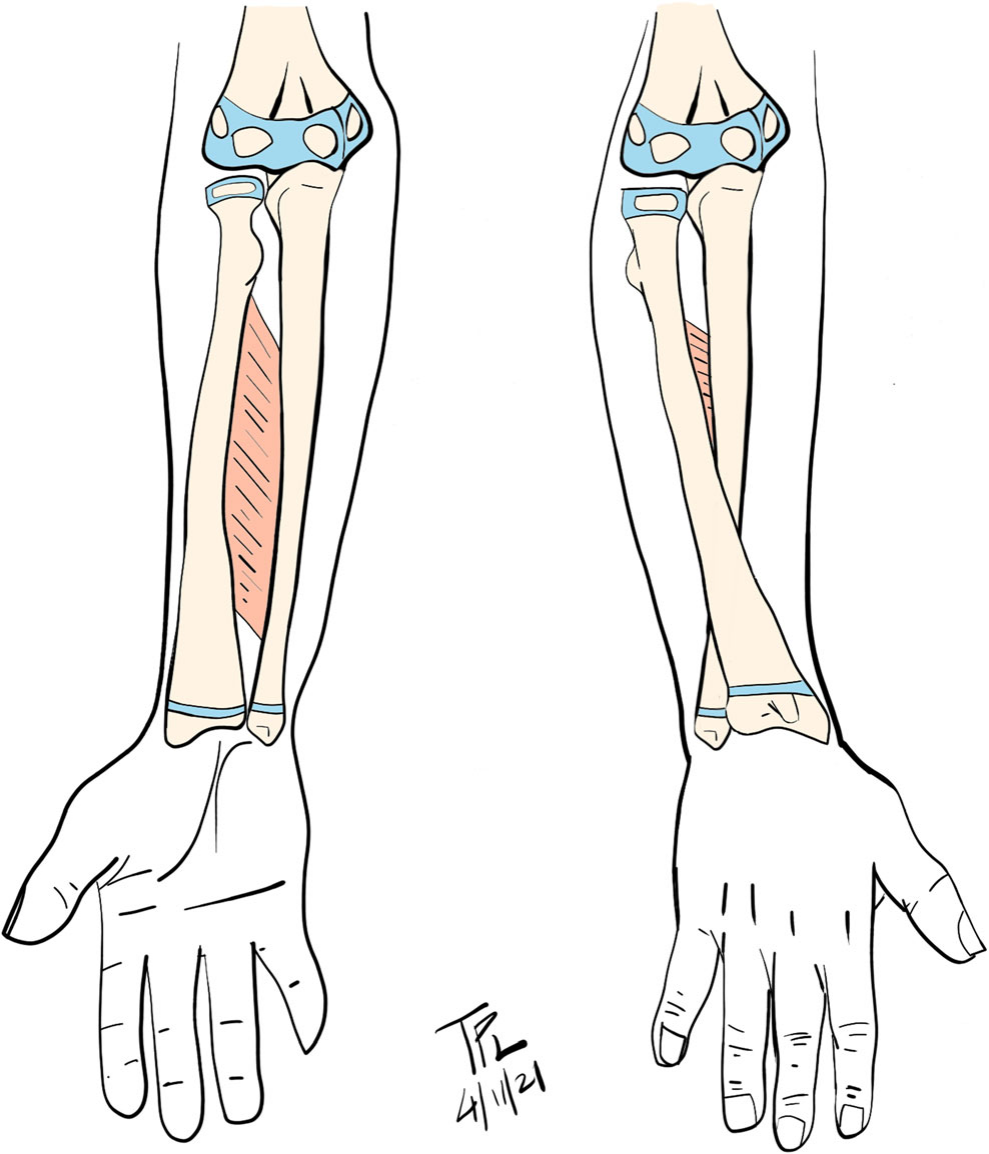
Interosseous membrane is a stabilizer of both forearm bones. The radius rotates around ulna during pronosupination. (Courtesy of T. Peter Li, MD, PhD)

The forearm consists of a relatively straight ulna and a curved radius that rotates about the ulna to achieve pronosupination at the proximal and distal radioulnar joints [23]. Growth plates at the proximal and distal ends of the bones contribute to growth and correlate with remodeling potential, with the distal physes contributing to the majority of longitudinal growth [24]. In fact, the distal physis contributes 75% of growth for the radius and 80% for the ulna [25]. The interosseous membrane traverses between the radius and ulna and acts as a stabilizer and force transmitter between the bones to decrease loads on the radiocarpal joint [23] (Fig. 1). Normal forearm pronation is approximately 71° and normal supination approximately 85° [17, 26].

Pediatric forearm fractures generally heal well with non-operative management, and less than anatomic reduction is often acceptable secondary to the child’s ability to remodel even substantial deformities due to the growth remaining prior to skeletal maturity. For this reason, most pediatric both-bone forearm fractures are treated non-operatively with closed reduction and casting and yield excellent functional results [6, 15, 27–29]. Nevertheless, fractures that occur in the midshaft or further proximal have the least amount of remodeling potential, as they are further away from the more active distal growth plates [2]. Displaced midshaft fractures also occur at the site of the greatest interosseous distance and can thus cause abnormal mechanics and impingement that can impair forearm rotation [30].

Given these constraints in the midshaft forearm, authors have attempted to define criteria for acceptable deformity in pediatric forearm fractures through anatomic, biomechanical, imaging, and clinical studies. While the scientific methodology of these studies is not robust, these values are based on the location of the fracture, the age of the patient remaining growth potential, and amount of deformity remaining after reduction [15–18]. The following are generally considered “acceptable” following closed reduction and casting of pediatric midshaft both bone forearm fractures (Table 1).

**Table 1:** Criteria of acceptable forearm fracture reduction

Deformity	Severity	Age (years)	Fracture position	Citation(s)
Radial malrotation	≤ 45^o^	≤ 16	-	[4]
Angulation (coronal *or* sagittal plane)	< 30^o^	≤ 10	Middle third	[7]
	< 10^o^	> 10	Middle third	[4, 8, 28]
	< 20^o^	≤ 14	Distal third	[1]
Displacement	100%	< 10	-	[4]
Bayonet apposition	< 1 cm	< 14	-	[24]
Axis deviation	< 5%	< 16	Middle and distal thirds	[27]

Morrey and colleagues [31] showed that 50° each of pronation and supination were sufficient for carrying out activities of daily living. Therefore, a combined 100° forearm arc of motion is considered functional. Previous studies have found that pediatric patients are able to compensate for forearm pronation deficit up to 60° by shoulder abduction [24, 31]. Nevertheless, concerns regarding physical appearance and long-term consequences along with the availability of modern techniques that enable near-anatomic deformity correction drive the effort to surgically correct even marginally acceptable deformities in the acute setting [17]. Deciding on how to correct the deformity can be challenging, given that the plane of deformity is frequently oblique and there is a complex interplay of angulation, translation, and fracture position contributing to the combined effect of forearm pronosupination defect. Younger and colleagues showed that the metric of axis deviation <5% (defined as the proportional displacement between fractured site and the anatomic axis relative to the full length of the anatomic axis) correlated better with restricted forearm pronosupination than angulation of fracture and fracture position [27]; however, its role in guiding treatment for correction of malunion is unclear.

Pediatric forearm fractures that do not meet the above-outlined criteria and go on to symptomatic malunion may be offered surgery to avoid poor functional and cosmetic results.

## Case

**Fig. 2:**
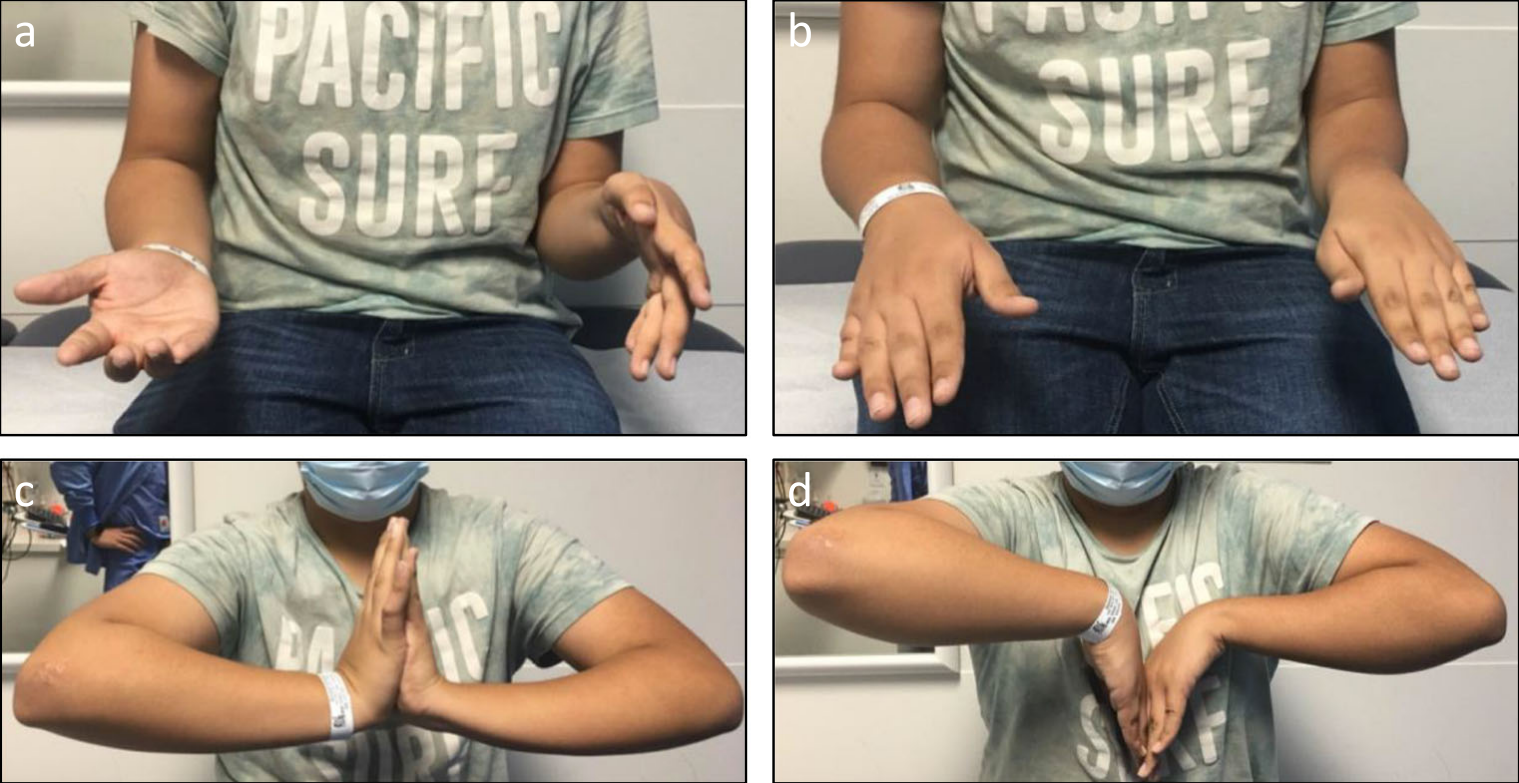
Preoperative clinical photographs of 15-year-old boy with limited left forearm supination and wrist flexion. **a** Maximal supination. **b** Maximal pronation. **c** Maximal wrist extension. **d** Maximal wrist flexion. (Courtesy of Sanjeev Sabharwal, MD, MPH)

**Fig. 3:**
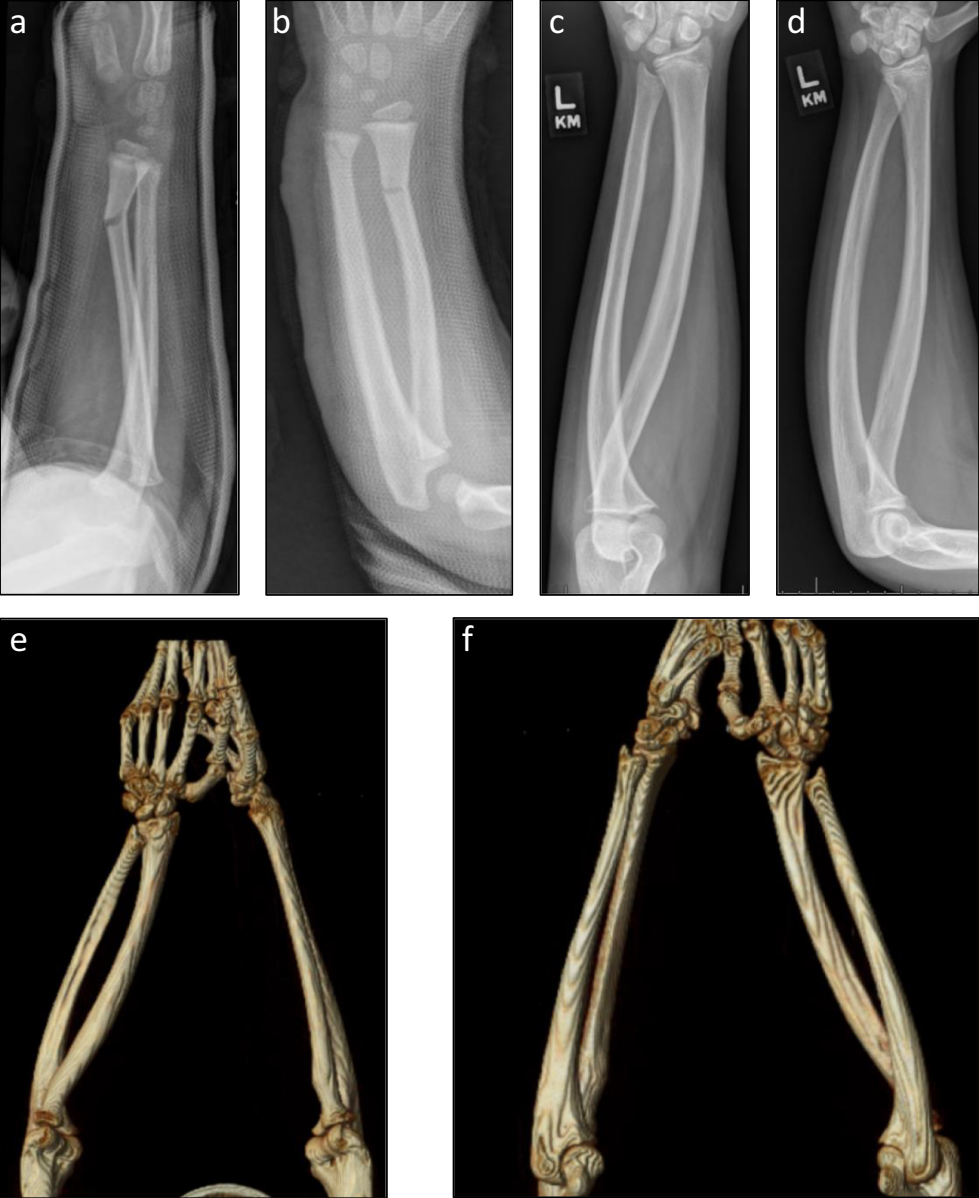
Preoperative imaging of patient in Fig. 2. **a–b** Orthogonal radiographs of left forearm after closed reduction and long arm casting at 3 years of age. **c–d** Radiographs of affected forearm at 15 years of age. **e** Three-dimensional rendering of CT of both forearms viewed from cephalad to caudad. **f** Same rendering viewed from caudad to cephalad. (Courtesy of Sanjeev Sabharwal, MD, MPH)

*A 15-year-old right-hand-dominant male presented to clinic with a chronic painless left forearm deformity and inability to perform certain daily functions and recreational sports such as catching a football. He had limited left forearm supination at −10° (right 90°) and wrist flexion at 45° (right 90°) (Fig.* *2**). He had sustained a distal forearm fracture when he was 3 years old and was treated in a long arm cast (Fig.* *3**a, b). Radiographs and 3D rendering of cross-sectional imaging confirmed a severe rotational malunion of his left forearm (Fig.* *3**c–f). This is an unusual case of forearm malunion since the overall alignment noted in the injury films at age 3 years are within acceptable limits.*

### Timing of Deformity Correction

Re-displacement risk is 7% after initial closed manipulation and casting. For impending malunion up to 24 days after injury, re-manipulation and closed treatment is a safe option [32]. Timing of surgical intervention for pediatric forearm shaft fractures is made on a case-by-case basis; however, earlier intervention (< 12 months from date of injury) may minimize the chronic effects of malrotation or angular deformity and potentially achieve greater restoration of motion. As the pediatric patient generally has rapid healing potential, corrective osteotomy should be considered when malunion take-down and compression plating no longer appear feasible. After the fracture consolidates, in as short as 2–3 months depending on the age of the patient, surgery is recommended if residual deformity may exist after accounting for remodeling potential.

In a retrospective review of 27 patients who required corrective osteotomy for loss of motion, those managed within 12 months of initial injury gained over twice the motion (79° vs. 30°) compared to those who underwent surgery greater than 12 months from initial injury [13]; the mean age of patients in the early treatment group was 14 (range, 9–17) years, while the mean age of the later treated patients was 12 (range, 4–26) years. The authors hypothesized that earlier intervention may avoid soft tissue scarring and interosseous membrane contracture that can compromise the improved mobility following the osteotomy. Similarly, in a meta-analysis of 11 cohort studies (median age 11 years), corrective osteotomy performed less than 12 months from initial trauma was a predictor of superior functional outcomes (gain in motion of 93° versus 61°) [33]. This study also showed that patient’s age at time of osteotomy was a predictor of postoperative gain of forearm rotation (<13 years, 87° vs ⩾13 years, 68°).

### Preoperative Evaluation and Planning

Initial patient assessment includes clinical examination of wrist and elbow motion as well as proximal and distal radial-ulnar joint stability. Pronosupination measurements should be performed with the arm adducted to the side of the patient’s trunk. Large angular deformities may be assessed visually. Full-length AP and lateral radiographs of both forearms should be obtained. If remodeling potential cannot be ascertained based on the patient’s chronologic age, history, or exam, then radiographs to determine skeletal age and growth remaining can be helpful. For multiplanar injuries or rotational deformity, a CT scan with the forearm in maximal pronation and supination can be considered. CT scans may also be used for emerging 3D computer-assisted planning with patient-specific instrumentation. The degree of anticipated correction can be based on clinical and radiographic measurements from the contralateral extremity.

If radiographs suggest significant torsional deformity, cross-sectional CT [13] or MRI [14] can be helpful to determine the amount of derotational correction needed. There is no evidence that cross-sectional CT [34] is superior to MRI for assessing torsional profiles. As MRI minimizes radiation exposure, it is the preferred advanced imaging modality in pediatric patients unless a CT scan is needed for obtaining patient-specific 3D models and instrumentation.

## Case

**Fig. 4:**
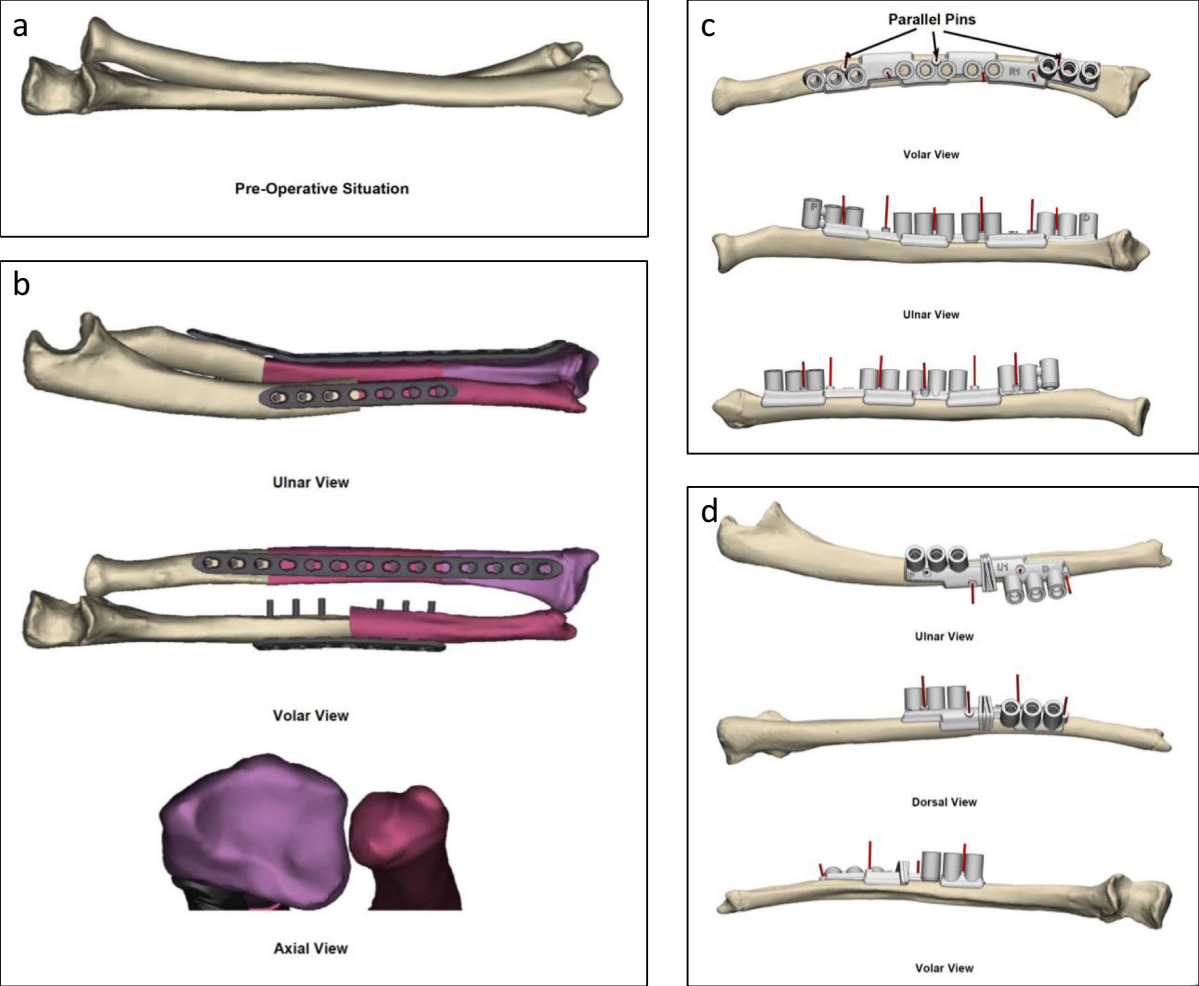
Preoperative planning to make patient-specific instrumentation for patient in Fig. 2. **a** Three-dimensional model of malunited forearm. **b** Intended osteotomy, correction, and internal fixation guided by uninjured forearm model. **c** Patient-specific guide for two-level osteotomy to correct the radius. **d** Patient-specific guide for single-level osteotomy to correct the ulna. (Courtesy of Sanjeev Sabharwal, MD, MPH)

*Given the complexity of this patient’s deformity which involves a combination of angular and rotational malunion, we performed osteotomies of the radius and ulna using patient-specific instrumentation. Select images demonstrating the preoperative plan based on CT scans using the uninjured forearm as the template (Fig.* *4**a, b). Patient-specific cutting guides are rapid-prototyped for performing a 2-level osteotomy of the radius (Fig.* *4**c) and a single osteotomy of the ulnar shaft (Fig.* *4**d).*

### Surgical Options

The goals of surgically correcting forearm fracture malunion include restoring forearm rotation, reducing pain, and correcting visually apparent deformities. Surgical options vary in technical complexity ranging from minimally invasive drill osteoclasis to open osteotomy assisted by 3D-planned patient-specific instrumentation. Since Dr. Mercer Rang and colleagues’ seminal report in 1984 of drill osteoclasis to correct forearm fracture malunion [10], many alternative options have been published. This section is not a comprehensive review of all available surgical techniques, but is meant to highlight different surgical options for which outcomes have been documented in the peer-reviewed literature.

### Percutaneous Drill Osteoclasis

Osteoclasis is the manual manipulation of the bone at the original fracture site with the intent to correct a nascent malunion [35, 36]. Osteoclasis through percutaneous drill holes (drill osteoclasis) enables a more controlled manual osteoclasis than conventional technique [10]. This technique relies on the presence of a thick periosteal sleeve that is often present in children and can help maintain the postoperative correction in a well-molded cast or with augmented stability with percutaneous wire fixation.

#### Advantages and Disadvantages

Advantages of this technique include minimal scarring, ability to correct severe angular deformity, and avoidance of internal fixation and an obligate second procedure to remove the hardware under anesthesia. Corrective power in restoring forearm rotation is unclear as preoperative rotation was not reported in the only series available [10].

#### Technique

Based on anatomic constraints, a small incision is made overlying the prominence of the maximally angulated portion of the individual bone(s), and a drill guide is brought down to periosteum at the site of the malunion. An appropriately sized drill bit with appropriate soft-tissue protection is used to create several holes through bone, focusing on the convexity of the deformity. This allows for controlled osteoclasis through manipulation under fluoroscopic guidance and effectively creates a greenstick fracture that can be reduced in acceptable alignment. Postoperatively, the forearm is immobilized in a well-molded long arm cast for 3–6 weeks. Weekly radiographic follow-up is recommended in the early postoperative period. When casting alone is unlikely to hold the fracture reduction, the surgeon should prepare for internal fixation. According to a study from early 1980s, authors reported a 27% prevalence of additional intramedullary fixation of the ulna and 7% use of crossed Kirschner wires in the radius [10]. These can be removed 6–24 weeks postoperatively, based on radiographic healing and implant used.

#### Outcome

Postoperatively, 67% of patients were satisfied, 73% regained full pronosupination, and 100% achieved angular correction to under 10° [10]. These patients were 5–15 years of age corrected at 2–10 months after initial injury.

### Open Osteotomy Using Conventional Techniques

Open osteotomy techniques for established malunions, especially with multiplanar deformities including both bones of the forearm, may require a more elaborate preoperative planning, considering not only angulation and rotation, but also length and translational deformity of the radius and ulna. Published techniques are based on preoperative and intraoperative radiographs or fluoroscopy [13, 14, 19, 20], with or without preoperative advanced cross-sectional imaging such as CT [13] or MRI[14].

#### Advantages and Disadvantages

This technique is not limited by skeletal maturity. However, it requires greater surgical exposure, longer operative time, and internal fixation which often requires a second surgery for removal.

#### Technique

**Fig. 5:**
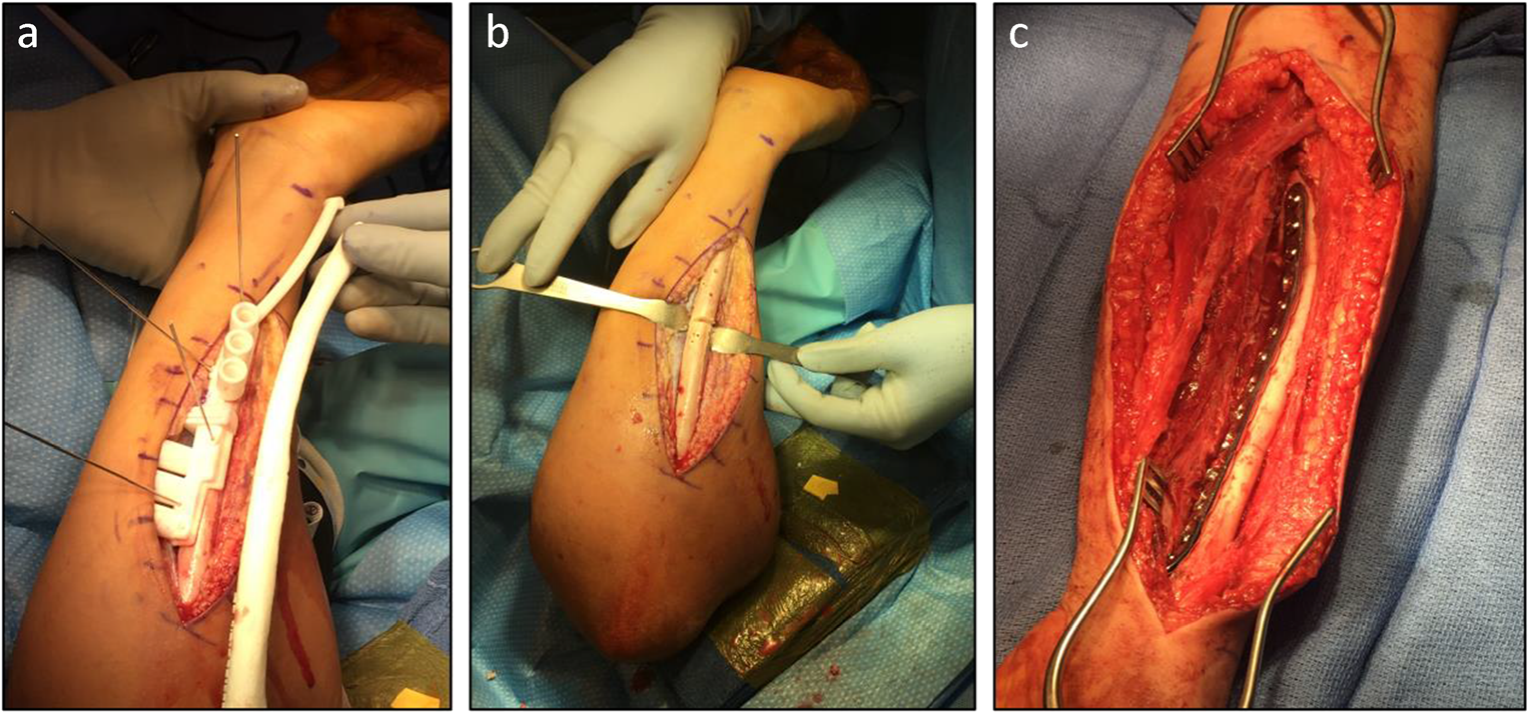
Intraoperative photographs demonstrating osteotomy using patient-specific cutting guide for patient in Fig. 2. **a** Patient-specific ulnar cutting guide held to patient’s ulna by wires; a plastic model of patient’s ulna can be used as an additional confirmation of guide placement accuracy. **b** Ulnar drill holes and osteotomy prior to reduction and internal fixation. **c** Volar view of internally fixed two-level radial osteotomy. (Courtesy of Sanjeev Sabharwal, MD, MPH)

Planning for deformity correction planes is facilitated by clinically examining and imaging both forearms [13]. The ulna is typically approached subcutaneously between the extensor carpi ulnaris and the flexor carpi ulnaris. The radius is typically approached through the volar approach of Henry. The order of which bone to correct is up to the surgeon’s preference, though for both-bone corrections, the ulnar osteotomy is typically performed first [13, 19, 20], especially in cases with limited supination, as it is technically difficult to position the forearm for a volar approach without first performing the ulnar osteotomy (Fig. 5).

Techniques using radiographs and intraoperative fluoroscopy rely on determining the plane of maximum angulation and performing a closing wedge osteotomy at the apex of angulation, followed by derotation to restore the anatomic relationships of the radial tuberosity, radial styloid, coronoid process, and ulnar styloid [19, 20]. Some surgeons recommend opening wedge osteotomy with interpositional structural bone graft if there is predictable shortening that would produce >1-mm ulnar variance [14]. Plate contouring and interpositional autogenous bone-grafting can be helpful if osteotomized fragments fail to achieve close bony apposition [13]. In cases of severe deformity, shortening of either or both forearm bones may be needed to reduce soft tissue tension [19]. Internal fixation can be achieved with either compression plating or intramedullary fixation; intramedullary fixation is usually reserved for skeletally immature patients [19]. Ideally, plate fixation should be secured by at least four cortices both proximal to and distal to the osteotomy, with care taken to avoid neurovascular and physeal injury.

One group of authors recommended release of the interosseous membrane in cases of pronosupination deficit refractory to corrective osteotomy and derotation [14]; however, this is controversial given the theoretical risk of synostosis [13]. The DRUJ should be assessed intraoperatively after the osteotomy is stabilized, particularly for patients whose surgical indication is painful DRUJ instability. Unstable DRUJ refractory to corrective osteotomy can be stabilized with imbrication of the palmar capsule and augmented with Kirschner wires [13].

*Postoperative management* varies widely. Some authors recommend postoperative immobilization with a sugar tong splint or long arm bi-valved cast exchanged for a better-fitting long arm cast at the first postoperative visit after swelling subsides for a total duration immobilization of 6 weeks [19]. One group advocated for long-arm splinting and continuous passive motion machine under physiotherapist’s supervision immediately postoperatively [20]. Others recommend immobilizing the forearm in a resting splint for 6 weeks, starting active motion exercises within the first 2 weeks, and utilizing dynamic stretching splints along with passive motion and strengthening after postoperative week 6 [14]. Frequency of radiographic follow-up is per surgeon preference; one group reported performing radiographs at 6 weeks, 12 weeks, 6 months, and 12 months postoperatively [20]. Plate removal is recommended at least 18 months postoperatively to avoid refracture [20], but there is no consensus regarding the optimal timing of hardware removal.

#### Outcome

This technique of open osteotomy and plating can restore 20–160° degrees of forearm rotation, averaging 79 [13] to 98 degrees [20] if performed within 1 year after injury. The rate of improvement in DRUJ pain and instability is 83% [13] to 100% [14]. Nearly complete angular correction can be expected up to 100% of patients [19]. It is important to note that the ability to achieve excellent outcomes is much lower if time from injury to corrective surgery is > 12 months [13, 20] or if the patient’s age is >10 years at the time of corrective surgery [20]. Interestingly, age at the time of injury, level of fracture, whether the radius or both bones were fractured, and whether the radius or both bones were osteotomized did not affect gain in forearm ROM in two retrospective case series [13, 20].

### Open Osteotomy Using Patient-Specific Instrumentation (3D-Planned)

Technological innovations including computer-simulated virtual surface modeling and rapid prototyping have made patient-specific instrumentation such as custom drill guides, cutting guides, contoured plating, and structural bone substitutes viable options for deformity correction [37–40].

#### Advantages and Disadvantages

3D-planned osteotomies on average have been reported to be 32 min shorter than conventional osteotomy in operative time [22]. However, the associated cost of 3D planning and production of patient-specific instrumentation needs to be considered, with additional reported costs of up to $4300 per case which includes CT of both forearms, planning, production of patient-specific cutting guides, and two pre-contoured titanium plates [41]. The surgeon must also account for 2–4 h of planning time using virtual modeling in addition to 28 [22] to 48 days [42•] of processing time needed between preoperative CT scanning and surgery. Additional radiation from CT scanning both forearms is unavoidable for this technique; the long-term health consequences are unclear.

#### Technique

The key difference in the perioperative management between 3D-planned and conventional osteotomy lies in the preoperative planning phase. CT scans of both the malunited forearm and the unaffected forearm are obtained; slice thickness can vary from 0.45 to 1.25mm [22, 42•, 43, 44]. It is unclear whether MRI affords adequate resolution for precise planning. Preoperative 3D-planning is achieved in two steps: (1) virtually planning the osteotomy and (2) designing and prototyping the patient-specific instrumentation. Virtual planning requires conversion of CT image data to virtual surface models in commercial software, e.g., Materialise Mimics Surgicase (Leuven, Belgium) [41, 45], Orthree Bone Viewer Bone Simulator (Osaka, Japan) [42•], or Kitware Visualization toolkit (Clifton Park, New York) [44]. Virtual osteotomies can then be simulated to achieve ideal correction of angular and/or torsional deformity [41] using the mirror-imaged unaffected forearm model as the normal template. Designing the patient-specific instrumentation can be done using various commercial software packages. For example, custom drill and cutting guides can be designed in Materialise 3-Matic (Leuven, Belgium) [41, 45] or CASPA [22, 46], and subsequently prototyped using medical grade polyamide or resin by 3D printing, which can be done on industrial grade 3D printers on site (Eden250, Objet Geometries, Rehovot, Israel; Viper si2, 3D systems, Rock Hill, South Carolina) [42•, 44] or be outsourced to one of various different companies (Materialise, Leuven, Belgium; Medacta International, Castel San Pietro, Switzerland; Sirris, Charleroi, Belgium; Amitek Prototyping, De Meern, Netherlands) [22, 41, 43–46]; custom-contoured plates can be designed in Mobelife (Leuven, Belgium) [41] and manufactured using titanium by metal 3D printing LayerWise (Leuven, Belgium). These instruments and implants are then verified on 3D-printed replicas of malunited and corrected forearm bones. Notably, the Food and Drug Administration in the USA has approved orthopaedic use of 3D-printed guides by Materialise (Leuven, Belgium) for patients at least 7 years of age; usage in younger age group is off-label.

The exposure of the ulna and radius is carried out in the usual fashion. Proper fit of the custom drill and cutting guides is verified intraoperatively by direct visualization and fluoroscopy after positioning them on the malunited bony surface. Drill holes are created via the custom drill guide, followed by osteotomy via the custom osteotomy guide with a micro-oscillating saw [41]. In certain available systems, initial reduction is performed and held by placing the custom plate using 2 smooth pegs in the most proximal and distal predrilled holes, followed final fixation using four non-locking fully threaded cortical screws, providing a total of six cortices of fixation both proximal to and distal to the site of osteotomy [41, 45]. Of course, custom-contoured plating is optional if the bony surface post-correction will allow proper fit of standard plating or if the surgeon plans to manually contour the plate [42•, 43, 44, 46].

## Case

### Intra-operative Photographs Demonstrate Correction and Internal Fixation Guided by Patient-Specific Instrumentation (Fig. 5)

*Postoperative management* varies. Reported protocols range from no immobilization to 2 weeks of either long arm cast [41] or splint immobilization followed by home exercise program under parental supervision [41]. Full load bearing and contact sports are restricted until osseous union is confirmed [41]. Plate removal was performed between 50 [22] and 100% [41] of patients; some surgeons routinely planned for removing hardware in skeletally immature patients [41], whereas other surgeons only removed hardware only if it became symptomatic [22] in skeletally mature patients.

### Outcome

For painful DRUJ instability, pain relief was reported in 100% of patients [41, 45]. The average improvement in supination ranged from 42 [41] to 52° [44]; the average improvement in pronation ranged from 19 [41] to 22° [44]. In studies that only reported total forearm pronosupination arc, the average improvement was from 41 [22] to 57° [45]. It is poorly understood whether time after injury matters for outcome in 3D-planned osteotomy, as it has not been directly addressed in published literature to date.

## Case

**Fig. 6:**
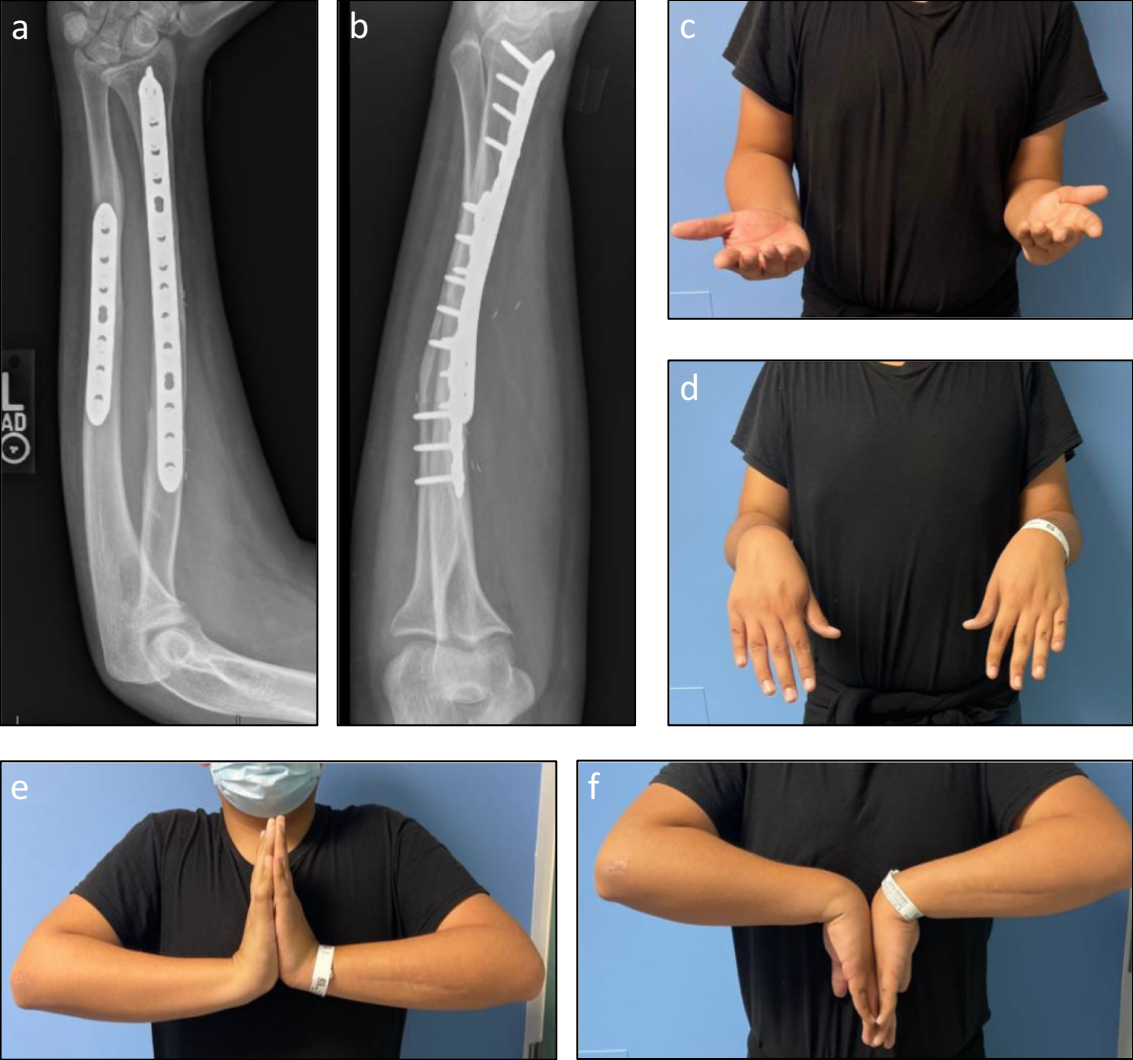
Seven months postoperative follow-up of patient in Fig. 2. **a–b** Orthogonal radiographs of corrected forearm with residual dorsal prominence of the distal ulna which is currently asymptomatic. **c** Maximal supination. **d** Maximal pronation. **e** Maximal wrist extension. **f** Maximal wrist flexion. (Courtesy of Sanjeev Sabharwal, MD, MPH)

*Radiographic and clinical follow-up at 7 months postoperatively show nearly full restoration of forearm supination and wrist flexion (Fig.* *6**). Dorsal prominence of the distal ulna is noted (Fig.* *6**b); he was asymptomatic at the distal radio-ulnar joint and resumed all activities, including football.*

### Autogenous Bone Graft and Substitutes

Grafting with autologous bone or structural bone substitute has been reported to facilitate bony contact in opening wedge derotational osteotomy whether 3D-planned or not. Use of autogenous cancellous bone grafting was noted in 50–100% of cases [13, 22, 42•, 46]. Bone grafting is often performed when an open wedge osteotomy is required [22, 46]. The autogenous bone graft is usually obtained from the iliac crest [22, 46], although small amounts of cancellous bone can be harvested locally, such as from the proximal ulna.

Despite the use of cancellous autogenous bone graft, nonunion after corrective osteotomy using patient-specific instrumentation can be as high as 20% [22, 41]. In some cases when one of both bones undergoes closing wedge osteotomy and the other bone requires an opening wedge, the removed wedge of bone can be fashioned to support the opening wedge [41]. In cases when this is not possible, there is no known report to date using structural bone allograft for correction of pediatric forearm malunion. In a series of 5 patients using patient-specific hydroxyapatite structural bone substitute implant [40], 100% achieved union within 5 months. While this incurs an additional cost to the procedure, it provides an alternative to freehand osteotomy of cortical bone allograft to produce the ideal structural support.

### Outcomes

The goals of improving forearm range of motion, pain in the distal radioulnar joint, and correcting visually apparent deformities can be achieved by the methods described above. To date, no single modality has been proven superior to others in addressing pediatric forearm malunion.

### Complications

Despite good to excellent results reported in the literature following surgical correction of pediatric forearm malunions, complications have been described including residual deformity, nonunion, infection, and damage to surrounding soft tissues. Reported rates of ulnar nonunion range 8–20% [22, 41], transient sensory loss of superficial branch of radial nerve up to 11%, hypertrophic scar up to 5%, and extensor pollicis longus weakness up to 5% [45]. Related to residual deformity, there are cases reported of worsened pronosupination after correction, refractory to therapy that required revision surgery [47]. Synostosis and ossification of the interosseous membrane can contribute to this stiffness [13]. A large, acute correction also presents a risk for compartment syndrome and neuropraxia due to stretch [48]. Surgeons acutely correcting a large deformity may choose to perform prophylactic fasciotomies or closely monitor the patient for neuro vascular compromise in the immediate postoperative period.

## Conclusions

When treating acute pediatric forearm fractures, the surgeon should pay careful attention to alignment and rotation, and consider the remodeling potential of the residual deformity at the fracture site for any given age in order to avoid a malunion that can cause long-term consequences. Forearm diaphyseal fracture malunion may occur from inadequate remodeling or substantial malreduction particularly in rotation, or extreme positioning of the forearm with cast immobilization that is not fully appreciated in the acute phase of treatment.

When malunion does occur and becomes symptomatic, either an osteoclasis (for nascent malunion) or an osteotomy can enhance function, reduce pain, and improve visible appearance of the deformity. Results are often dependent on several factors such as the patient’s age, magnitude and location of deformity, chronicity of malunion, preoperative planning method, surgical technique, and implant(s) used. Selection of the ultimate surgical option depends on available resources, surgical indication, and surgeon expertise.

Emerging techniques can potentially enhance the ease, speed, and accuracy of performing corrective osteotomy using patient-specific instrumentation. However, it remains unclear whether the change in clinical outcome justifies the additional cost and radiation exposure associated with these emerging technologies. Prospective clinical studies with robust methodology are needed to compare the outcome and complication rates between the traditional approach and 3D-planned patient-specific techniques.
